# Optical Force of Bessel Pincer Light-Sheets Beam on a Dielectric Sphere of Arbitrary Size

**DOI:** 10.3390/nano12213723

**Published:** 2022-10-23

**Authors:** Shu Zhang, Bing Wei, Qun Wei, Renxian Li, Shiguo Chen, Ningning Song

**Affiliations:** School of Physics, Xidian University, Xi’an 710071, China

**Keywords:** Bessel pincer light-sheets, GLMT, optical force, Maxwell’s stress tensor, angular spectrum expansion method

## Abstract

In the framework of Generalized Lorenz–Mie theory (GLMT), based on the expansion results of electromagnetic field radiation components of Bessel pincer light sheets beam acting on dielectric particles of arbitrary size, the expression of radiation force components in a Cartesian coordinate system is obtained by using the Maxwell stress tensor method. On the one hand, the effects of the refractive index and the equivalent radius of spherical particles on the distribution of radiation force are discussed; On the other hand, the influence of beam scaling parameter and beam order of Bessel pincer light sheets beam on the distribution of radiation force are investigated. The results indicate that the changes of particle’s refractive index and effective radius only affect the distribution of radiation force. However, the beam scaling parameter and beam order of Bessel pincer light sheets beam have a very sharp impact on the convergence position, distribution range and bending degree far away from the wave source of the radiation force. Single-beam optical tweezers using the self-focusing and self-bending Bessel pincer light-sheets beam are crucial for applications such as single molecule biophysics, optical manipulation and particle separation/clearing.

## 1. Introduction

The optical scattering, extinction and absorption cross section characterize the strength of light-matter interaction. They quantify the fraction of power a particle scatters, extincts, or absorbs. Although the optical force is another one to characterize the interaction between target and electromagnetic wave. Optical forces stem from momentum transfer directly between photons and objects, or indirectly between surrounding media and objects when light interacts with objects. Optical trapping [1] (due to strongly focused beam on the object), optical pulling [2] (due to scattering force) and optical binding [3] (Multiple scattering between particles) are there typical beam types. We are interested in the type of optical trapping for a monochromatic beam—optical tweezer. Optical tweezers are exquisite position and force transducers. Therefore, they are widely used for high-resolution measurements in fields as varied as physics [4,5], biology [6,7,8,9], optical lift [10,11], all-optical wavelength routing [12], optical scalpels and scissors [13,14,15], optical stochastic cooling [16,17], optical force sensor [18], Dynamic-Microscopy [19], and materials science [20]. The forces exerted by carefully sculpted wavefronts of light [21] offer precisely the level of access and control needed for rapid progress across all of these fields.

Since the discovery that a comet’s tail points away from the sun, theoretically explained by Maxwell (1883) [22], experimentally proved by Lebedev [23] and Nichols and Hull [24], light processes have been known to exert optical radiation pressure. The optical tweezer [25,26], first proposed by Arthur Ashkin and coworkers at AT&T Bell Laboratories, has been a classical tool for optical manipulating and trapping of micrometer-sized particles. Particle characteristics and light source characteristics of optical tweezers are the key factors in the operation of optical tweezers. The techniques used for manipulating microparticles rely on the electric dipole interaction energy [27,28], which scales down approximately with particle volume. Thus, thermal fluctuations are large enough to overwhelm the trapping forces at the nanoscale [29]. The method of discrete dipole approximation [30,31,32] is the typical one to manipulate particles using the dipole model. However, it is limited to the particle size, whose size is close to the wavelength ($\left|mkd\right|<$1, *m* is the refractive index, *k* is the wave number and *d* is the distance of dipole). Mie–Debye theory [33], is applicable to these large particles (larger than the wavelength). The Rayleigh method [34] is a universal one to those small particles (smaller than the wavelength). The above method is confined to electromagnetic calculation of a single size (compared to wavelength). Theoretical expressions for the internal and external electromagnetic fields of a homogeneous spherical particle illuminated by a focused laser beam based upon a rigorous, complete solution of Maxwell’s equations have been developed, each utilizing different approaches, by Kim and Lee [35] Gouesbet, Maheu, and Grehan [36] and Barton, Alexander, and Schaub [37]. They can be used to calculate optical force on the particles of arbitrary size. Bardon et al. [38] derived series expressions for net radiation force and net torque for a spherical particle by an arbitrary defined monochromatic beam. This provides a common method for the optical force calculating using the Maxwell stress tensor [39,40], under the steady-state condition. The optical force depends not only on the property of the target itself but also on the configuration of the incident beam [41]. Light waves carry energy and momentum. If light is scattered or absorbed by a particle, its momentum is changed, and by conservation of momentum, it exerts a force on the particle called radiation pressure.

To illustrate the trapping of optical force, it is significant to describe the wavefront modification of the incident beam. A Gaussian beam [42] is one of the classical and early optical tweezers’ sources. The focusing characteristics of a Gaussian beam have universal applications [32,43] in optical manipulation. However, it is easily limited by diffraction in long-distance scattering. Bessel beams [44,45,46,47,48,49] with self-reconstruction and non-diffraction characteristics break through the distance limit. Airy beams [50,51], with the properties of the acceleration transverse and non-diffraction, supports the bending angles along the propagation direction, and thus the Airy beam is convenient for particle clearing [52], optical force switching [53] and Optical Force-Fluorescence [41]. Recently, the two-dimensional illustration of living tissue is one of important researches to reveal the characteristics of biological tissue, such as in optofluidics [54] involving particle surface analyses, and light-based imaging modalities [55] in biomedicine to the refinement and technical improvement of light-sheet imaging. Mitri has done many works in light-beams in Airy light-sheets [56,57], Bessel–Gaussian light-sheets [58], fractional Bessel-–Gauss pincers light-sheets [59], using the methods of analytical method and discrete dipole approximation(DDA). However, the bending angle of Airy beam is relatively small. The larger bending angles and auto-focusing are needed in some situations, such as in particle manipulation applications with minimal hampering by obstacles. The Bessel pincer, first proposed by Mitri, exhibits a negative force by a higher-order Bessel beam in the context of acoustical/scalar waves. Bessel pincer light-sheet beams [60] provide enhanced autofocusing and curving/bending capabilities. In our method [61], the slice-section with a ‘thin sheet’ of Bessel pincer light-sheets beam is considered. Dielectric materials [62,63] are widely used in target electromagnetic scattering [64,65] because of their dielectric properties [66,67] under an electric field.

According to the given components of the incident electric field and scattering field in ref. [61], the Maxwell stress tensor is obtained. Subsequently, the net force on the particle can be determined by integrating the dot product of the outwardly directed normal unit vector and Maxwell stress tensor over a surface enclosing the particle. The influences of the Bessel pincer light-sheets (mainly focusing on beam order and scaling parameter) acting on a dielectric sphere particle (mainly focusing on the refractive index and dimensionless particle size), will be discussed.

## 2. Methods

### 2.1. Angular of Bessel Pincer Light-Sheets Beam

**Figure 1:**
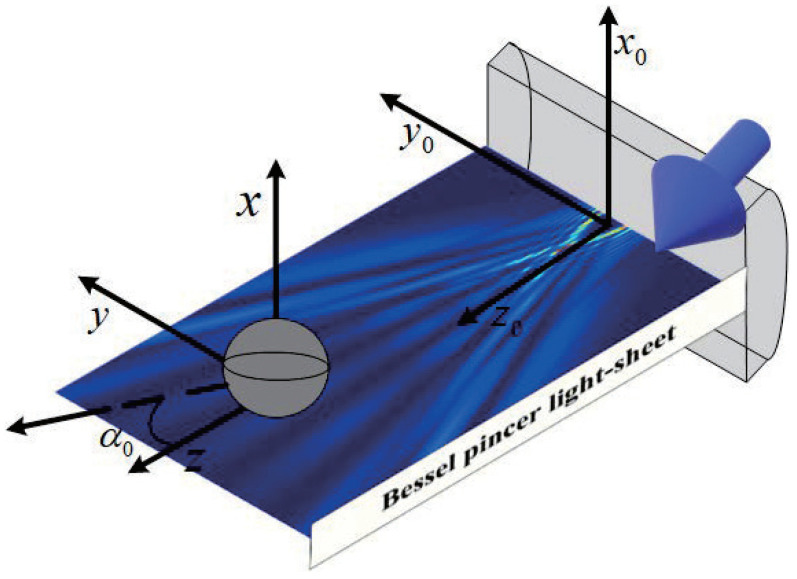
Distribution of radiation force component $F_{z}$ under the illumination of Bessel pincer light-sheets beam(λ = 0.6328 μm, $\alpha_{0}$ = 0.5 and *l* = 10) on the dielectric sphere (radius is 0.1 μm).

Considering the TE polarization of Bessel pincer light-sheets beam, the angular spectrum of Bessel pincer [60] is as below:

(1) $$\begin{array}{cc} S_{x}\left(p,q\right) & =\left(\frac{kE_{0}}{2\pi }\right)\int_{-\infty }^{+\infty }J_{l}\left(\alpha ky\right)e^{-ikpy}dy\\ \; & =\frac{E_{0}i^{-1}}{k\alpha_{0}^{l}\sqrt{\alpha_{0}^{2}-p^{2}}}ℜ\left\{{\left(p+i\sqrt{\alpha_{0}^{2}-p^{2}}\right)}^{l}\right\} \end{array}$$

where, $\alpha_{0}$ is the beam scaling parameter; *ł* is the beam order; (*p*, *q*) is the directional cosines (p=sinα and q=cosα), and α is the angle of propagation of the individual plane wave. Assuming the dielectric sphere is illuminated by the Bessel pincer light-sheets beam, as shown in Figure 1, and the the location of the wave source is at $\left(y_{0},z_{0}\right)=\left(0,0\right)$. Through the multipole expansion of the incident plane wave and considering the orthogonal property of vector spherical wave function, the series expression of incident plane wave is obtained.

(2) $$E_{i}=-\sum_{n=1}^{\infty }\sum_{m=-n}^{n}iE_{mn}\left[p_{mn}N_{mn}^{\left(1\right)}\left(kr\right)+q_{mn}M_{mn}^{\left(1\right)}\left(kr\right)\right]$$

where,

(3) $$E_{mn}=i^{n}\left|E_{0}\right|{\left[\frac{2n+1}{n(n+1)}\frac{(n-m)!}{(n+m)!}\right]}^{1/2}$$

(4) $$M_{mn}^{\left(1\right)}\left(kr\right)=\left[i\pi_{mn}\left(\cos\theta \right)e_{\theta }-\tau_{mn}\left(\cos\theta \right)e_{\phi }\right]j_{n}\left(kr\right)e^{im\phi }$$

(5) $$\begin{array}{c} N_{mn}^{\left(1\right)}\left(kr\right)\;\;\;\;\;=\left[\tau_{mn}\left(\cos\theta \right)e_{\theta }+i\pi_{mn}\left(\cos\theta \right)e_{\phi }\right]\frac{1}{kr}\frac{d}{dr}\left[rj_{n}\left(kr\right)\right]e^{im\phi }\\ \;\;\;\;\;\;\;\;\;\;\;\;\;\;\;\;\;\;\;\;\;\;\;\;\;\;\;\;\;\;\;\;\;\;\;\;\;\;\;\;\;\;\;+e_{r}n\left(n+1\right)P_{n}^{m}\left(\cos\theta \right)\frac{j_{n}\left(kr\right)}{kr}e^{im\phi } \end{array}$$

with

(6) $$p_{mn}=k\frac{i^{1-m}}{E_{0}}D_{mn}^{1/2}\int_{\alpha =0}^{\pi /2}d\alpha e^{-ik\cos\alpha z_{0}}S_{x}\left(\alpha \right)\pi_{mn}\left(\cos\alpha \right)\cos\alpha$$

(7) $$q_{mn}=k\frac{i^{1-m}}{E_{0}}D_{mn}^{1/2}\int_{\alpha =0}^{\pi /2}d\alpha e^{-ik\cos\alpha z_{0}}S_{x}\left(\alpha \right)\tau_{mn}\left(\cos\alpha \right)\cos\alpha$$

(8) $$D_{mn}=\frac{\left(2n+1\right)\left(n-m\right)!}{n(n+1)(n+m)!}$$

(9) $$\pi_{mn}\left(\cos\theta \right)=\frac{m}{\sin\theta }P_{n}^{m}\left(\cos\theta \right)\;$$

(10) $$\tau_{mn}\left(\cos\theta \right)=\frac{dP_{n}^{m}\left(\cos\theta \right)}{d\theta }$$

where $P_{n}^{m}\left(\cos\alpha \right)$ represents the associated Legendre polynomials of degree *n* and order *m*, $j_{l}\left(\cdot \right)$ is the spherical Bessel function of *l* order, and $e_{\left(r,\theta ,\phi \right)}$ are radial, polar and azimuthal unit vectors, respectively.

### 2.2. Optical Force on a Sphere

Following the previous work, we broaden the scattering of a Bessel pincer light-sheets beam to optical force. The net radiation force can be determined by integrating the dot product of the outwardly directed normal unit vector $\hat{r}$ and Maxwell’s stress tensor [35,68] over a surface enclosing the particle.

(11) $$F=\oint_{S}\hat{r}\cdot \langle \overset{↔}{\;T}\rangle dS$$

where, *S* is arbitrary closed surface surrounding the particle, and $\hat{r}$ is the unit normal vector at arbitrary point on the surface.$\overset{↔}{\;T}$ is the Maxwell stress tensor, writen as

(12) T↔=12ReεEE*+μHH*−12εE·E*+μH·H*I↔

where, $\overset{↔}{\;I}$ is the unit tensor, and *є* and *μ* are the permittivity and permeability coefficient of particles, respectively. For the loss-free environment and unbounded closed surface, the Cartesian component of the optical force acting on a spherical particle is below,

(13) Fx=ReF1,Fy=ImF1,Fz=ReF2

where,

(14) $$\begin{array}{c} \begin{array}{c} F_{1}=\frac{2\pi \varepsilon_{0}}{k^{2}}|E_{0}{|}^{2}\sum_{n=1}^{\infty }\sum_{m=-n}^{n}\left\{\begin{array}{c} \frac{{\left[\left(n-m)(n+m+1\right)\right]}^{\frac{1}{2}}}{n(n+1)}\\ \times \left(\begin{array}{c} {\tilde{a}}_{m,n}{\tilde{b}}_{m+1,n}^{*}+{\tilde{b}}_{m,n}{\tilde{a}}_{m+1,n}^{*}\\ -{\tilde{p}}_{m,n}{\tilde{q}}_{m+1,n}^{*}-{\tilde{q}}_{m,n}{\tilde{p}}_{m+1,n}^{*} \end{array}\right) \end{array}\right.\\ -{\left[\frac{n(n+2)(n+m+1)(n+m+2)}{{\left(n+1\right)}^{2}\left(2n+1\right)\left(2n+3\right)}\right]}^{\frac{1}{2}}\times \left(\begin{array}{c} {\tilde{a}}_{m,n}{\tilde{a}}_{m+1,n+1}^{*}+{\tilde{b}}_{m,n}{\tilde{b}}_{m+1,n+1}^{*}\\ -{\tilde{p}}_{m,n}{\tilde{p}}_{m+1,n+1}^{*}-{\tilde{q}}_{m,n}{\tilde{q}}_{m+1,n+1}^{*} \end{array}\right)\\ \left.+{\left[\frac{n(n+2)(n-m)(n-m+1)}{{\left(n+1\right)}^{2}\left(2n+1\right)\left(2n+3\right)}\right]}^{\frac{1}{2}}\times \left(\begin{array}{c} {\tilde{a}}_{m,n+1}{\tilde{a}}_{m+1,n}^{*}+{\tilde{b}}_{m,n+1}{\tilde{b}}_{m+1,n}^{*}\\ -{\tilde{p}}_{m,n+1}{\tilde{p}}_{m+1,n}^{*}-{\tilde{q}}_{m,n+1}{\tilde{q}}_{m+1,n}^{*} \end{array}\right)\right\} \end{array} \end{array}$$

and

(15) $$\begin{array}{c} \begin{array}{c} F_{2}=-\frac{4\pi \varepsilon_{0}}{k^{2}}|E_{0}{|}^{2}\sum_{n=1}^{\infty }\sum_{m=-n}^{n}\left\{\frac{m}{n(n+1)}\times \left({\tilde{a}}_{m,n}{\tilde{b}}_{m,n}^{*}-{\tilde{p}}_{m,n}{\tilde{q}}_{m,n}^{*}\right)\right.\\ \left.+{\left[\frac{n(n+2)(n-m+1)(n+m+1)}{{\left(n+1\right)}^{2}\left(2n+1\right)\left(2n+3\right)}\right]}^{\frac{1}{2}}\times \left(\begin{array}{c} {\tilde{a}}_{m,n}{\tilde{a}}_{m,n+1}^{*}+{\tilde{b}}_{m,n}{\tilde{b}}_{m,n+1}^{*}\\ -{\tilde{p}}_{m,n}{\tilde{p}}_{m,n+1}^{*}-{\tilde{q}}_{m,n}{\tilde{q}}_{m,n+1}^{*} \end{array}\right)\right\} \end{array} \end{array}$$

with

(16) $$\begin{array}{c} \begin{array}{c} {\tilde{a}}_{m,n}=a_{m,n}-\frac{1}{2}p_{m,n},\;\;\;\;\;\;\;\;\;\;\;\;\;{\tilde{p}}_{m,n}=\frac{1}{2}p_{m,n}\\ {\tilde{b}}_{m,n}=b_{m,n}-\frac{1}{2}q_{m,n},\;\;\;\;\;\;\;\;\;\;\;\;\;{\tilde{q}}_{m,n}=\frac{1}{2}q_{m,n} \end{array} \end{array}$$

*a_n_* and *b_n_* are the classical Mie coefficients [33]. Since the energy of the incident light is proportional to $|E_{0}{|}^{2}$ in Equation (11), the formula states that the net radiation force on the particle is proportional to the energy of the incident beam [35].

## 3. Results

This section mainly includes three parts: Firstly, compare and verify the simulation results under different numerical methods; Secondly, the influence of particle parameters (refractive index of sphere, partical radius) and beam parameters (beam scaling parameters $\alpha_{0}$ and beam order *ł*) on the force spatial distribution is analyzed; Thirdly, the trend of particle parameters (refractive index of sphere, partical radius) and beam parameters (beam scaling parameters $\alpha_{0}$ and beam order *ł*) with beam propagation position is discussed.

### 3.1. Numerical Validation

In this part, the simulation results based on different numerical methods are given. Initialization parameters are as follows: the incident wavelength of Bessel pincer light-sheets beam is 1.064 μm; the refractive index of the sphere is 1.59 and the ambient refractive index is 1.33; the radius of sphere is 0.01 μm; the scaling parameter $\alpha_{0}$ is equal to 0.1 and the beam order *ł* is 1.

#### 3.1.1. Incident Intensity

**Figure 2:**
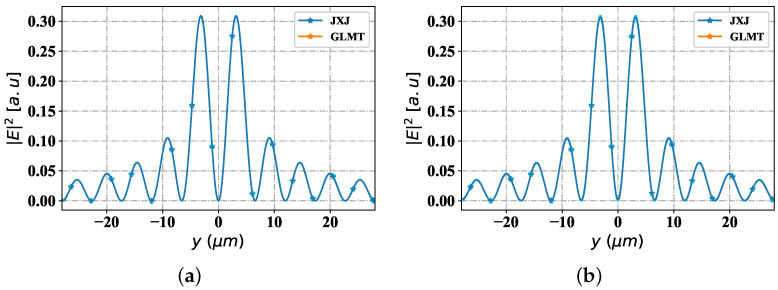
Validation of different ${\left|E\right|}^{2}$ with GLMT, and JXJ. (**a**) TE; (**b**) TM.

With all above parameters pre-determined, Figure 2 shows the change trend of the incident field intensity of Bessel pincer with the spatial position under two polarization modes (TE and TM), using analytical method (JXJ-blue curve plus Pentagram) (method in [60]) and reconstruction method (yellow curve plus Pentagram-the method used in this paper). With the change of the spatial distance y, the field intensity distribution trends of the incident field (corresponding to the two methods) have good consistency. Two numerical methods are used to supplement the verification of paper [59], which shows the effectiveness of the reconstruction method.

#### 3.1.2. Force Component of $f_{y}$ and $f_{z}$

**Figure 3:**
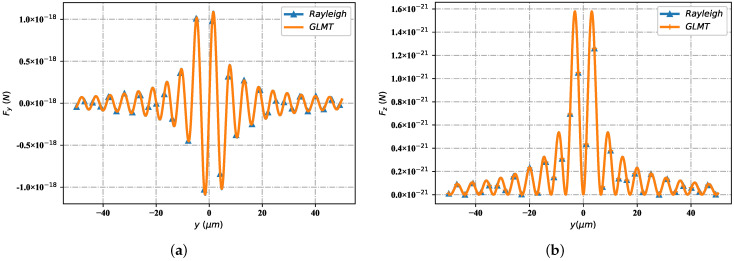
Validation of different $F_{y}$ and $F_{z}$ with Rayleigh and GLMT. (**a**) $F_{y}$; (**b**) $F_{z}$.

Furthemore, to clearly demonstrate the availability of optical force, the feasibility of the radiation force results is verified by comparing the calculation results of radiation force components of the Rayleigh method [44] and the Maxwell stress tensor method based on GLMT reconstruction. As shown in Figure 3a, it can be deduced from the line profile that the force component $F_{y}$ is in good agreement with both Rayleigh and GLMT (Maxwell stress tensor). The same is true of the force component $F_{z}$ (Figure 3b)—there is good consistency of the two methods.

### 3.2. Longitudinal and Transverse Optical Force $F_{y}$ and $F_{z}$

This part gives the radiation force components $F_{y}$ and $F_{z}$ in the yz plane with the change of spatial position, with emphasis on the influence of particle refractive index, particle equivalent radius (relative to wavelength), beam scaling parameter $\alpha_{0}$ and beam order *ł*. Assuming that the amplitude of the electric filed is equal to $E_{0}$ = $1.0\times 10^{6}$ V/m. The refractive index of environmental medium is defined as $m_{2}$ = 1.36 (ethylalcohol environment), and that of particle is $m_{1}$ = 1.5. The wavelength of incident Bessel pincer light-sheets beam is set to λ = 0.532 μm.

#### 3.2.1. Different Radius of the Particle

**Figure 4:**
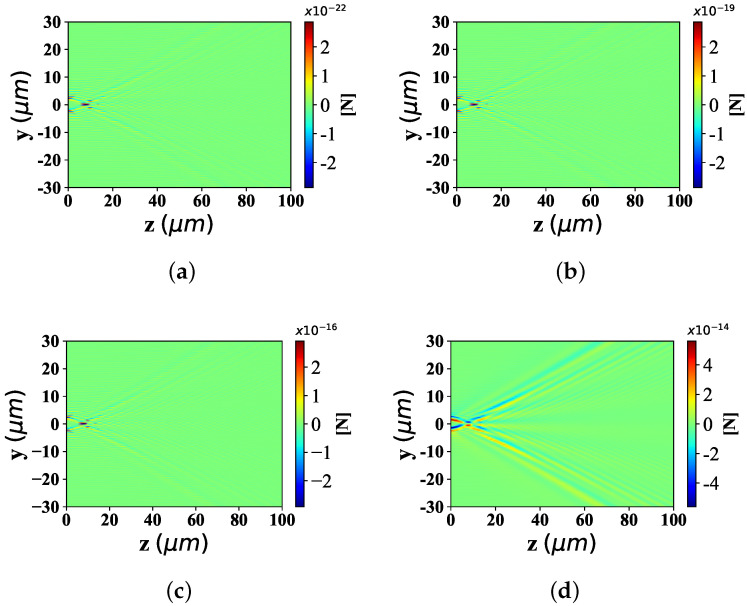
Distribution of $F_{y}$ under different spherical radius (radius is equal to 0.001 λ, 0.01 λ, 0.1 λ and λ, respectively) acting on Bessel pincer light-sheets beam (λ = 0.532 μm, $\alpha_{0}$ = 0.5, and *l* = 10). (**a**) radius = 0.001λ; (**b**) radius = 0.01λ; (**c**) radius = 0.1λ; (**d**) radius = 1λ.

Here, the spatial distribution of radiation force component $F_{y}$ with the change of particle equivalent radius, is illustrated in Figure 4. The beam scaling parameter $\alpha_{0}$ is 0.5 and beam order *ł* is 10. The radius of spherical particle is defined as 0.001 λ, 0.01 λ, 0.1 λ and 1 λ, respectively. Figure 4a displays the distribution of optical force component $F_{y}$, shows the moderate distribution range along the y-axis, the beam convergence point close to the wave source, and the bending angle with a certain expansion angle along the z−axis, far from the convergence point. Equally, Figure 4b shows the range of distribution along the y-axis with the same width as that in Figure 4a, the beam convergence point at the same location as in Figure 4a, and the bending angle with the same bending degree away from the convergence point as in Figure 4a. The distribution in Figure 4c,d is the same as that in Figure 4a. In particular, the values of the optical force component $F_{y}$ vary greatly. Specifically, the order of magnitude of $F_{y}$ in Figure 4a is smaller than that of the third power of 10 in Figure 4b, the fifth power of 10 in Figure 4c and the eighth power of 10 in Figure 4d.

**Figure 5:**
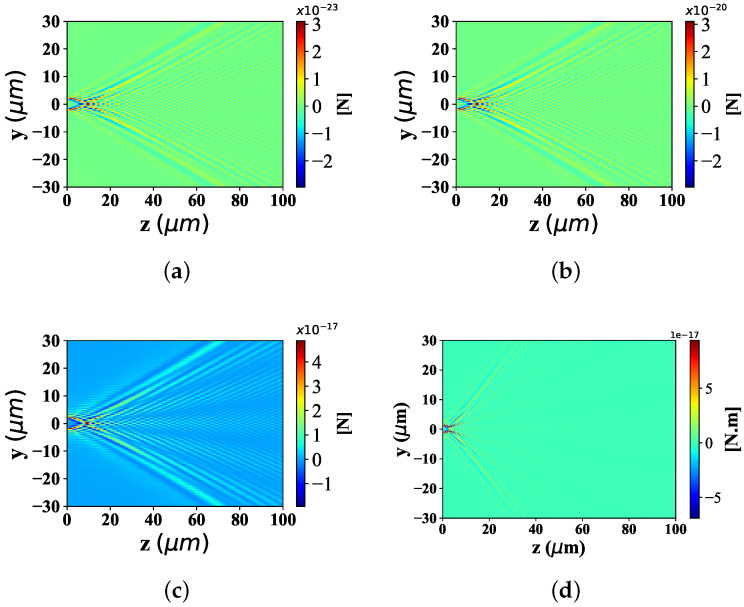
Distribution of $F_{z}$ under different spherical radius (radius is set to 0.001 λ, 0.01 λ, 0.1 λ and λ) acting on Bessel pincer light-sheets beam (λ = 0.532 μm, $\alpha_{0}$ = 0.5, and *l* = 10). (**a**) radius = 0.001λ; (**b**) radius = 0.01λ; (**c**) radius = 0.1λ; (**d**) radius = 1λ.

Figure 5 shows the longitudinal optical force component $F_{z}$ with spherical particles of different radius sizes. The simulation parameters are consistent with Figure 4. From Panel (a) to (d), the $F_{z}$ distribution range along y−axis, has the identical distribution range and location. The position of beam convergence point shall be consistent, and the bending degree of the beam away from the beam convergence point is almost the same. Similar to the distribution of optical component $F_{y}$ under different particle sizes, the magnitude of the $F_{z}$ value is the difference of optical force component $F_{z}$ distribution under different particle sizes. Moreover, the larger the particle radius is, the higher the corresponding $F_{z}$ value is.

#### 3.2.2. Different Beam Scaling Parameter $\alpha_{0}$ of Bessel Pincer Light-Sheets Beam

Here are the noticeable trends of the optical force component $F_{y}$ and $F_{z}$ in the y−z plane, with different beam scaling parameter $\alpha_{0}$ ($\alpha_{0}$ = 0.2, 0.5, 0.8). The beam order is *l* = 10 and the spherical radius is set to 0.1 μm.

**Figure 6:**
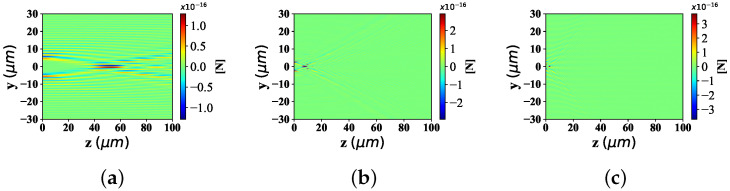
Distribution of $F_{y}$ under the interaction between the sphere and Bessel pincer light-sheets beam with different beam scaling parameter $\alpha_{0}$ ($\alpha_{0}$ = 0.2, 0.5, and 0.8; beam order is *l* = 10). (**a**) $\alpha_{0}$ = 0.2; (**b**) $\alpha_{0}$ = 0.5; (**c**) $\alpha_{0}$ = 0.8.

As can be seen in Figure 6, the distributions about optical force component $F_{y}$ are illustrated with different beam scaling parameters. Form the point of the trend of distribution range along the *y* -axis, Figure 6a has a fairly wide distribution range. There is a small distribution range in Figure 6b, which is much smaller than that in Figure 6a. Figure 6c retains some of the distribution range, but has the smallest distribution range among the three. From the perspective of beam convergence point position along *z*-axis, in Figure 6a, the distance between the convergence point of Bessel pincer light sheets beam and the source point of the wave source is dozens of wavelengths. In Figure 6b, the assembly position of the Bessel pincer light sheets beam is much closer to the wave source than that in Figure 6a. In contrast, the source position of Bessel pincer light sheets beam in Figure 6c is closer to the wave source than that in Figure 6b.

**Figure 7:**
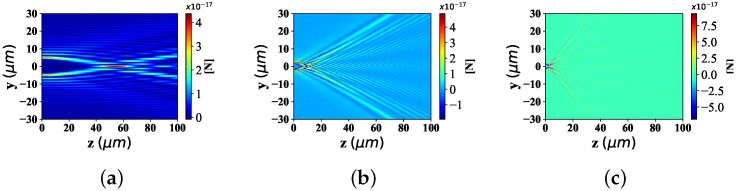
Distribution of $F_{z}$ under the interaction between the sphere and Bessel pincer light-sheets beam with different beam scaling parameter $\alpha_{0}$ ($\alpha_{0}$ = 0.2, 0.5, and 0.8; beam order is *l* = 10). (**a**) $\alpha_{0}$ = 0.2; (**b**) $\alpha_{0}$ = 0.5; (**c**) $\alpha_{0}$ = 0.8.

In Figure 7, the distribution of optical force component $F_{z}$ with different beam scaling parameter $\alpha_{0}$ can be seen. Similar to the perspective analysis in Figure 6, the distribution range of the optical component $F_{z}$ along the *z*-axis in Figure 7a, is the longest range, followed by Figure 7b,c which is the shortest of the three. Considering the distance between the convergence point of Bessel pincer light sheets beam and the wave source, the location of the beam convergence point in Figure 7a is the farthest from the wave source, followed by Figure 7b, and Figure 7a which is the closest among the three.

#### 3.2.3. Different Beam Order *l* of Bessel Pincer Light-Sheets Beam

**Figure 8:**
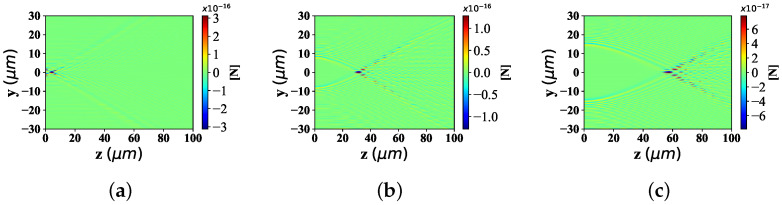
Distribution of $F_{y}$ under the interaction between the sphere and Bessel pincer light-sheets beam with different beam order *l* (*l* = 5, 45, 85; $\alpha_{0}$ = 0.5; radius is 0.1 μm). (**a**) *ł* = 5; (**b**) *ł* = 45; (**c**) *ł* = 85.

**Figure 9:**
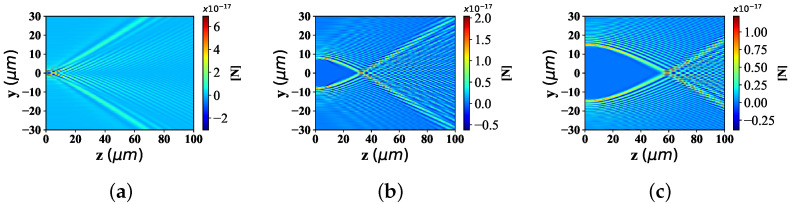
Distribution of $F_{z}$ under the interaction between the sphere and Bessel pincer light-sheets beam with different beam order *l* (*l* = 5, 45, 85; $\alpha_{0}$ = 0.5; radius is 0.1 μm). (**a**) *ł* = 5; (**b**) *ł* = 45; (**c**) *ł* = 85.

According to the Figure 8 and Figure 9, the changing trend of optical radiation force component $F_{y}$ and $F_{z}$ in yz space position (particle radius is 0.1λ, beam scale parameter is $\alpha_{0}$ = 0.5) with the change of beam order (even order is 6, 12, 26, and odd order is 5, 45, 85), is discussed.

Figure 8a–c give the distribution of radiation force component $F_{y}$ under different beam orders when the beam order *l* is odd. The distribution range of the radiation force component $F_{y}$ along the *y*-axis in Figure 8b is much wider than that in Figure 8a, and the location of the beam convergence point is farther from the location of the wave source than that in Figure 8a. Moreover, the bending degree of the beam far from the beam convergence point in Figure 8b is larger than the angle in Figure 8a. Further, when the radial force component $F_{y}$ is distributed along the y-axis, Figure 8c has a much wider range than that in Figure 8b; The position of the beam convergence point is farther than that in Figure 8b, and the scattering angle of the beam far away from the convergence point is more curved than that in Figure 8b.

The distribution of radiation force component $F_{z}$ under different beam orders, is discussed in Figure 9. Similar to the distribution of $F_{y}$ values in Figure 8, the variation range of $F_{z}$ number along the *y*-axis increases with the growing of beam order; The beam convergence point position of $F_{z}$ value ascends with the raising of the beam order *l*; However, the spreading degree of the Bessel pincer light-sheets beam (far from the convergence point), decreases with the increase of the beam order.

#### 3.2.4. Different Refractive Indices of the Particle

**Figure 10:**
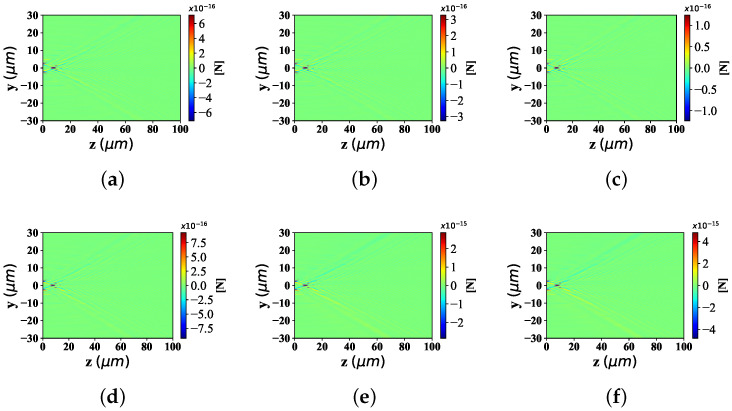
Distribution of $F_{y}$ under different refractive indic ($m_{1}$ = 1.0, 1.2, 1.3, 1.8, 2.8, 3.8) for the interaction of Bessel pincer light-sheets beam on the spherical particle. (**a**) $m_{1}$ = 1.0; (**b**) $m_{1}$ = 1.2; (**c**) $m_{1}$ = 1.3; (**d**) $m_{1}$ = 1.8; (**e**) $m_{1}$ = 2.8; (**f**) $m_{1}$ = 3.8.

**Figure 11:**
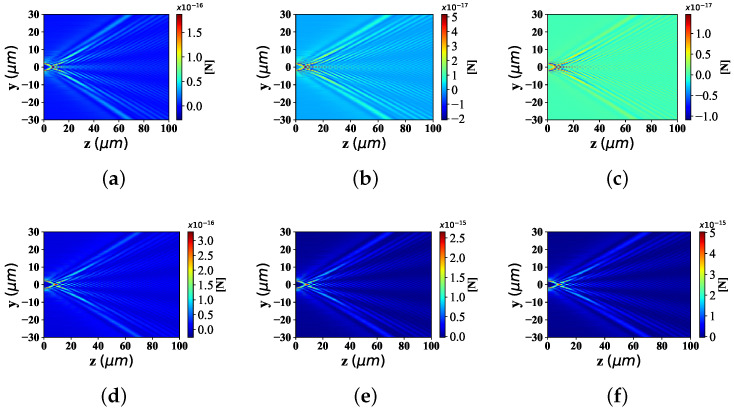
Distribution of $F_{z}$ under different refractive indices ($m_{1}$ = 1.0, 1.2, 1.3, 1.8, 2.8, 3.8) for the interaction of Bessel pincer light-sheets beam on the spherical particle. (**a**) $m_{1}$ = 1.0; (**b**) $m_{1}$ = 1.2; (**c**) $m_{1}$ = 1.3; (**d**) $m_{1}$ = 1.8; (**e**) $m_{1}$ = 2.8; (**f**) $m_{1}$ = 3.8.

The particle refractive index affects the absorption of beam particle interaction. The distribution of optical radiation force component $F_{y}$ and $F_{z}$ under the influence of particle refractive index, is shown in Figure 10 and Figure 11, including the part higher than the environmental refractive index ($m_{1}$ = 1.8, 2.8 and 3.8, respectively) and the part lower than the environmental refractive index ($m_{1}$ = 1.0, 1.2, and 1.3, respectively). The beam scaling parameter is $\alpha_{0}$ = 0.5, the beam order is *l* = 10, and the particle radius is 0.1 μm.

Figure 10 depicts the the distributions of optical force component $F_{y}$ under different refractive indices of the particle. For the case where the particle refractive index is less than the environmental refractive index, from Figure 10a–c, the range of the optical force component $F_{y}$ along the *y*-axis almost keeps the same width, which is different from the magnitude of the numerical value. The beam convergence points along the *z*-axis are basically at the same position; The beam spread angle distributed along the *z*-axis (far from the beam convergence point) keeps the same bending degree. On the other hand, when the particle refractive index is greater than the environmental refractive index, the radiation force range, beam convergence point position and beam bending degree (far from the beam convergence point) distributed along the y-axis are all at the same width, the same convergence point position and the same bending degree of the expansion angle from Figure 10d–f. However, only the numerical value of the optical radiation force is different. This is due to the same beam scaling parameter $\alpha_{0}$, beam order *l* and particle radius when the optical radiation force component $F_{y}$ is distributed under different particle refractive index.

The distribution of optical force component $F_{z}$ in Figure 11 resembles that in Figure 10. Nevertheless, in the radiation force distribution $F_{z}$ of particles with different refractive index below the environmental refractive index, the magnitude of the radiation force component $F_{z}$ increases with the raising of the particle refractive index. On the contrary, in the $F_{z}$ distribution of different particle refractive indices (higher than the environmental refractive index), the value of the radiation force component $F_{z}$ is generally greater than the $F_{z}$ distribution of different particle refractive indices (lower than the environmental refractive index).

### 3.3. $F_{y}$ and $F_{z}$ versus the Dimensionless Parameter ka

This subsection describes the variation trend of $F_{y}$ and $F_{z}$ with particle radius radius and refractive index $m_{1}$, beam order *ł* and beam scaling parameter $\alpha_{0}$ along the propagation direction ($r=\sqrt{y^{2}+z^{2}}$). The incident wave length remains the same as the simulation value in the previous section, and the amplitude of the electromagnetic field remains numerically constant.

**Figure 12:**
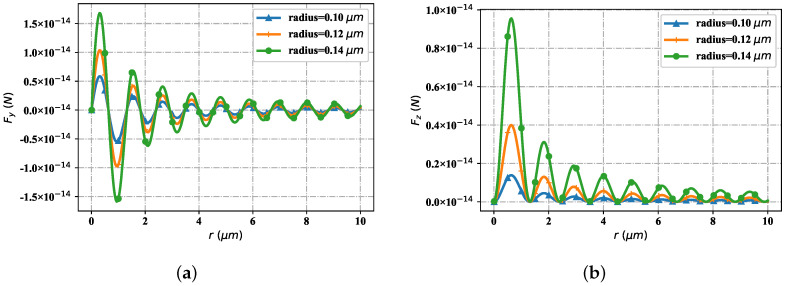
The implication of spherical radius for the changing of $F_{y}$ and $F_{z}$. (**a**) $F_{y}$ with radius; (**b**) $F_{z}$ with radius.

Figure 12 shows the distribution of optical force component $F_{y}$ and $F_{z}$ with different spherical radius (0.10 μm, 0.12 μm and 0.14 μm, respectively) along the propagation *r*. When *r* is equal to 10 μm, the curve gradually tends to converge. Therefore, it is sufficient to discuss the variation of optical force component at a propagation distance of 10 μm. Shown as Figure 12a, at the first stagnation point, $F_{y}$ with the particle radius of 0.1 μm is zero, $F_{y}$ with a particle radius of 0.12 μm and $F_{y}$ with a particle radius of 0.14 μm are also zero. At the first wave crest position, when the particle radius is 0.1 μm, the $F_{y}$ value is smaller than the $F_{y}$ value corresponding to the particle radius of 0.12 μm; The $F_{y}$ value corresponding to the radius of 0.12 μm is smaller than that corresponding to the particle radius of 0.14 μm. At the second stagnation point, the $F_{y}$ values corresponding to particle radii of 0.1 μm, 0.12 μm and 0.14 μm are all the same and zero. At the first wave crest, the $F_{y}$ value corresponding to the particle radius of 0.1 μm is greater than that corresponding to the particle radius of 0.12 μm; The $F_{y}$ value corresponding to 0.12 μm radius is greater than that of 0.14 μm particle radius. The position of the third stagnation point is the same as that of the second stagnation point. The $F_{y}$ values corresponding to different particle radii correspond to zero. At the position of the second wave crest, the distribution of $F_{y}$ values corresponding to the particle radius is the same as that at the positions of the second wave crest, but the values are smaller. The distribution of $F_{y}$ value corresponding to particle radius at the second wave trough is the same as that at the first wave trough, but the value is larger. It can be seen that when the distribution of the optical force component $F_{y}$ changes along the propagation direction, the value of $F_{y}$ corresponding to different particle radius has positive and negative values. At the same time, it reaches the maximum value at the peak position, the minimum value at the trough position and the zero value at the stagnation point position.

As shown in Figure 12b, at the first stagnation point, the $F_{z}$ value corresponding to the radius of all particles is zero. At the first wave crest, the $F_{z}$ value of particle radius 0.1 μm is smaller than the $F_{z}$ value corresponding to particle radius 0.12 μm; When the particle radius is 0.12 μm, the $F_{z}$ value is smaller than that corresponding to the particle radius of 0.14 μm. The $F_{z}$ distribution corresponding to the particle radius at the second stagnation point is the same as that at the first location ($F_{z}$ = 0). At the position of the second wave crest, the $F_{z}$ values corresponding to all particle radii are smaller than those at the position of the first wave crest, but the distribution of the $F_{z}$ values corresponding to particle radii is similar to that at the first wave crest. The $F_{z}$ value corresponding to the particle radius at the third, fourth and subsequent stagnation points is zero. The $F_{z}$ value corresponding to the particle radius at the third and subsequent wave crest gradually decreases, but the distribution is similar to that at the first wave crest. It can be seen that, when the optical force component changes along the direction of propagation, the values of $F_{z}$ corresponding to the different particle radii are greater than or equal to zero. Moreover, at the same time the maximum value at the peak position is reached, the station position at the same time reaches the value zero- there is no trough. In a word, the $F_{y}$ component is distributed with the particle radius, showing staggered distribution of wave crest, stagnation point and wave trough. The $F_{y}$ value at the wave crest is larger with the increase of particle radius, and the overall $F_{y}$ value decreases gradually with the increase of the number of wave crests; The $F_{y}$ value at the wave trough is smaller with the increase of particle radius, and the overall $F_{y}$ value gradually increases with the increase of the number of wave peaks. The $F_{z}$ component is distributed alternately with the wave crest and the stationary point as the particle radius increases. The $F_{z}$ value at the wave crest increases as the particle radius increases, and the overall value decreases gradually with the number of wave crests.

**Figure 13:**
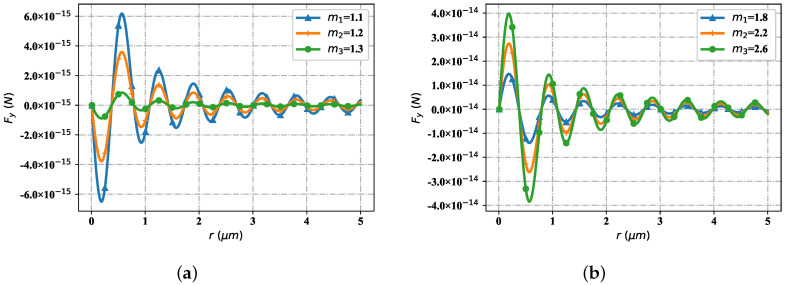
The distribution of $F_{y}$ under different refractive indices, including the lower indices and upper indices (comparing to the surrounding index). (**a**) $F_{y}$ with low indices; (**b**) $F_{y}$ with high indices.

Figure 13 shows that the spatial distribution trend of the optical radiation force component $F_{y}$ with the change of particle refractive index. Ambient refractive index is $m_{2}$ = 1.36, considering two cases: the particle refractive index is less than the environmental refractive index and the particle refractive index is greater than the environmental refractive index. As shown in Figure 13a (the particle refractive index is smaller than the environmental refractive index), at the first stagnation point, the $F_{y}$ values of different refractive indexes tend to zero. At the first wave crest position, when the particle refractive index is 1.1, the $F_{y}$ value is smaller than the $F_{y}$ value corresponding to the particle refractive index of 1.2; When the particle refractive index is 1.2, the $F_{y}$ value is smaller than the $F_{y}$ value corresponding to the particle refractive index of 1.3. At the second stagnation point, the $F_{y}$ values corresponding to particles with different refractive indexes are all zero. At the first wave trough position, when the particle refractive index is 1.1, the $F_{y}$ value is greater than the $F_{y}$ value corresponding to the particle refractive index of 1.2. At the third and all other stagnation points, the $F_{y}$ values of particles with different refractive index are zero. At the second wave crest, the distribution of $F_{y}$ values of different particle refractive indices is similar to that at the first wave crest, but the overall value is smaller than that at the first wave crest. The distribution of $F_{y}$ value of the particle refractive index at the second wave trough is similar to that of the particle refractive index at the first wave trough, but the overall $F_{y}$ value is larger. At the third wave crest and the wave crest after it, the distribution of $F_{y}$ values of different refractive indexes is similar to that at the second wave crest, but the overall value is smaller. At the third and subsequent wave troughs, the distribution of $F_{y}$ values of different refractive indexes is similar to that at the second wave trough, but the overall value gradually increases. As shown in Figure 13b (the particle refractive index is all greater than the environmental refractive index), the distribution trend of $F_{y}$ values corresponding to different particle refractive indexes is similar to that in Figure 13a, but the overall value is an order of magnitude higher. With the increase of the refractive index of different particles, the $F_{y}$ value corresponding to the refractive index of different particles at the wave crest gradually increases, the $F_{y}$ value corresponding to the refractive index of different particles at the stagnation point is zero, and the $F_{y}$ value corresponding to the refractive index of different particles at the wave trough gradually decreases.

**Figure 14:**
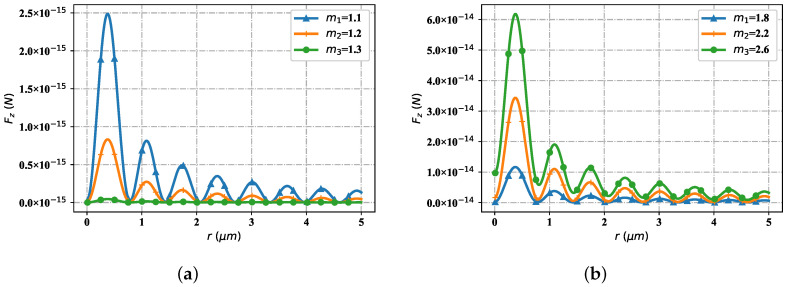
The distribution of $F_{z}$ under differet refractive indices, including the lower indices and uper indices. (**a**) $F_{z}$ with low indices; (**b**) $F_{z}$ with uper indices.

Figure 14 contains the change of particle refractive index, but the spatial distribution trend of the optical radiation force component $F_{z}$. Similarly, consider two cases: the particle refractive index is less than the environment refractive index and the particle refractive index is greater than the environment refractive index. As shown in Figure 14a (the particle refractive index is smaller than the environmental refractive index), at the first stagnation point, the particle $F_{z}$ values of different refractive indexes are all zero. At the first wave crest, when the particle refractive index is 1.1, the $F_{z}$ value is smaller than the corresponding $F_{z}$ value when the particle refractive index is 1.2; The $F_{z}$ value when the particle refractive index is 1.2 is smaller than the corresponding $F_{z}$ value when the particle refractive index is 1.3. All $F_{z}$ values at the second and subsequent stagnation points tend to zero. At the second wave crest, the distribution trend of $F_{z}$ values corresponding to particles with different refractive index is similar to that at the first wave crest, but the overall $F_{z}$ values are smaller than those of particles at the first wave crest. At the third and subsequent wave peaks, the distribution trend of $F_{z}$ values of particles with different refractive indexes is similar to that at the second wave peak, but the overall values gradually decrease. As shown in Figure 14b (the particle refractive index is greater than the environmental refractive index), the change trend of $F_{z}$ values corresponding to particles with different refractive index is similar to that in Figure 14a, but the overall $F_{z}$ value is one order of magnitude higher. At the stagnation point, the $F_{z}$ values corresponding to different particle refractive indices tend to zero; At the wave crest, the corresponding $F_{z}$ value gradually increases with the increase of the particle refractive index value; The further it moves back, the smaller the $F_{z}$ values corresponding to different particle refractive indices at the wave crest become, but the changing trend remains unchanged.

**Figure 15:**
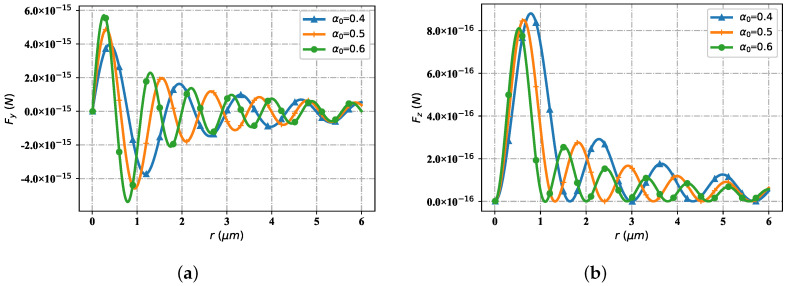
The influence of beam scaling parameter $\alpha_{0}$ for optical force component $F_{y}$ and $F_{z}$. (**a**) $F_{y}$ with different $\alpha_{0}$; (**b**) $F_{z}$ with different $\alpha_{0}$.

Figure 15 shows the variation trend of the optical force components $F_{y}$ and $F_{z}$ interacting with particles and beams in the space propagation area with different beam parameters (beam order l=1, radius is eauql to 0.1 μm). Through simulation tests, when the spatial position is selected as *r* = 6 μm, the change of optical force component tends to converge under different beam parameters. Figure 15a shows the distribution trend of the optical force component $F_{y}$ with different beam scaling parameters. At the first stagnation point, for different scale parameters $\alpha_{0}$, the value of $F_{y}$ tends to zero. When beam scaling parameter $\alpha_{0}=0.6$, the first wave crest appears in the value of $F_{y}$; When beam scaling parameter $\alpha_{0}$ = 0.5, the value of $F_{y}$ exhibits a second peak, but the value is smaller than the first peak, and it is farther from the source point of the light source than the first peak; Further, when the beam scaling parameter $\alpha_{0}$ = 0.4, the value of $F_{y}$ exhibits a third peak, which is smaller than the second peak, and is farther from the source point of the light source than the second peak. When the value of $F_{y}$ becomes negative with the beam scaling parameter $\alpha_{0}$, the value of $F_{y}$ corresponding to $\alpha_{0}$ = 0.6 first reaches the minimum value of the wave trough; The second is beam scaling parameter $\alpha_{0}$. The value of $F_{y}$ corresponding to $\alpha_{0}$ = 0.5 becomes the minimum value of the second wave valley. The value of $F_{y}$ is larger than that of the first wave valley, and the position is farther from the source point of the light source than that of the first wave valley; When the beam parameter is $\alpha_{0}$ = 0.4, the corresponding value of $F_{y}$ becomes the third wave valley, which is larger in value than the corresponding value of $F_{y}$ at the second wave valley, and is farther from the source point of the light source than the second wave valley. The second group (the fourth, fifth and sixth) wave crest and its subsequent peak digit value and location distribution are similar to those of the first group (the first, second and third), but the overall value becomes smaller. Figure 15b displays the distribution trend of the optical force with different beam scaling parameters, but with respect to the component $F_{z}$ (all $F_{z}$ values are greater than or equal to zero). Near the source point of the light source, the value of $F_{z}$ corresponding to different beam scale parameters tends to zero. When the $F_{z}$ value corresponding to $\alpha_{0}$ = 0.4 reaches the first wave peak, the $F_{z}$ value corresponding to $\alpha_{0}$ = 0.5 reaches the wave peak, but the $F_{z}$ value is smaller than the value of $\alpha_{0}$ = 0.4, and the position of the wave peak is closer to the position of the wave source. When the $F_{z}$ value corresponding to $\alpha_{0}$ = 0.6 reaches the first wave peak, the $F_{z}$ value corresponding to $\alpha_{0}$ = 0.5 is smaller, but closer to the position of the wave source. For $F_{z}$ corresponding to $\alpha_{0}$ = 0.4, $\alpha_{0}$ = 0.5 and $\alpha_{0}$ = 0.6, the situation when it reaches the second peak is similar to that when it reaches the first peak, but the overall value is smaller. The $F_{z}$ value of $\alpha_{0}$ = 0.4, $\alpha_{0}$ = 0.5 and $\alpha_{0}$ = 0.6 respectively reach the third peak, the fourth peak and the subsequent peak, and so on until convergence.

**Figure 16:**
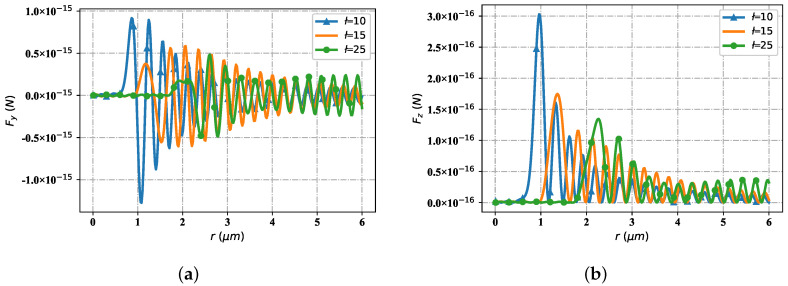
The influence of beam order *l* for optical force component $F_{y}$ and $F_{z}$. (**a**) $F_{y}$ with different *ł*; (**b**) $F_{z}$ with different *ł*.

Figure 16 represents the spatial distribution of the optical radiation force component when the Bessel pincer light sheets beam interacts with particles as the beam order changes (particle radius is 0.1 μm, beam scaling parameter $\alpha_{0}$ = 0.5, incident wave length is λ = 1.064 μm, beam order *l* is 10, 15 and 25). When the spatial position is *r* = 25 μm, the distribution of radiation force component tends to converge. Figure 16a shows the distribution of the radiation force component $F_{y}$ with the change of spatial position with different beam orders. The wave crest of $F_{y}$ curve with beam order of *l* = 10 first reaches the first peak near the wave source; The next step is the $F_{y}$ curve with the beam order of *l* = 15. Relative to the curve with beam order of *l* = 10, the peak position is reached at the second place with a smaller peak but far from the wave source. When the $F_{y}$ curve with beam order of *l* = 10 and *l* = 15 alternately reaches its corresponding peak, the curve with beam order of *l* = 25 reaches its first peak position (the second peak position of the $F_{y}$ curve with the relative to beam order of *l* = 15) for the first time. Then, the $F_{y}$ curves corresponding to the three beam orders interlace with each other in turn and reach the subsequent peak positions respectively. In general, for the $F_{y}$ curve with beam order *l* = 10, the value of $F_{y}$ corresponding to the wave crest is higher than that of the $F_{y}$ curve with beam order *l* = 15, and the value of $F_{y}$ curve with beam order *l* = 25 is the lowest among the three. On the contrary, in the trough period, the value of $F_{y}$ curve with beam order of *l* = 25 is the highest among the three, followed by $F_{y}$ curve with beam order of *l* = 15, and finally by $F_{y}$ curve with beam order of *l* = 10. Figure 16b shows the radiation force component $F_{z}$, which changes with the spatial location distribution under the influence of beam order. With the staggered distribution of wave crests, the $F_{z}$ components are numerically greater than or equal to zero. When the $F_{z}$ curve of beam order *l* = 10 first reaches the first wave peak at a position close to the wave source with a large value; The $F_{z}$ curve with the beam order *l* = 15 reaches the first wave peak with a smaller value at the position far from the wave source. When the $F_{z}$ curve with the beam order *l* = 15 reaches the third peak, the $F_{z}$ curve with beam order *l* = 25 starts to reach its first peak (numerically smaller than the first peak of the $F_{z}$ curve with beam order *l* = 15). Then, the peak value of each order successively reaches the corresponding peak until convergence.

## 4. Conclusions

The result reported above was obtained by using GLMT which is based on the Maxwell stress tensor method, and electromagnetic field expression. We discussed two aspects: one is the influence of spherical particle parameters, the other is the implication of Bessel Pincer light-sheets beam parameters. By showing the results of the pseudo-color map and comparing the results of the line graph, we acknowledge that the change of particle parameters affects the magnitude of the corresponding numerical value of the radiation force component. Furthermore, the change of beam parameters affects the distance of radiation force convergence position (from the wave source), and the degree of beam divergence and bending (after the distance from the wave source position). According to the control variable method, for the particle parameters, the value of the radiation force increases with the growing of the particle refractive index and the equivalent radius. As for beam parameters, with the improving of beam order *l*, the distribution range of radiation force along the *y*-axis gradually expands, the beam convergence location is farther and farther away from the wave source, and the bending degree away from the wave source convergence location gradually presents a small angle; On the contrary, with the amplifying of beam scale parameters $\alpha_{0}$, the distribution range of radiation force along the *y*-axis gradually narrows, the beam convergence location is closer to the wave source location, and the bending degree away from the wave source convergence location gradually presents a larger angle. The result presented here opens a new landscape of possible phenomena for anatomy of biological cells in two dimensions, including particle rotating and sorting devices, optical sectioning, imaging microscopy and particle characterization and sizing.

## Data Availability

Some or all data, models, or code generated or used during the study are available in a repository or online in accordance with funder data retention policies (Provide full citations that include URLs or DOIs.)

## Abbreviations

The following abbreviations are used in this manuscript:

GLMT	Genralized Lorenz Mie Theory
